# Supplementary material for: Predicting the Textural Properties of Plant-Based Meat Analogs with Machine Learning
Source: Foods. 2023 Jan 11;12(2):344. doi: 10.3390/foods12020344 (PMC9858592; doi:10.3390/foods12020344)
Supplement: Supplementary file 1 [file foods-12-00344-s001.zip › foods-2115553-supplementary.pdf]

Supplementary Document

S1) Descriptive statistics of the curated dataset in this study.

Table S1: Descriptive statistics of our curated dataset.

	Features							Response Variables	
	**protein**	**target moisture**	**moisture**	**ash**	**carbs**	**fat**	**fiber**	**Hardness**	**Chewiness**
mean	70.02	60.45	5.79	4.38	13.43	3.81	2.38	44.91	35.88
std	9.90	6.55	1.96	0.84	5.37	3.08	3.49	22.54	17.06
min	56.00	43.67	2.00	3.37	2.90	0.20	0.02	14.63	12.34
25%	63.18	56.49	4.86	3.95	9.20	0.20	0.51	23.79	19.99
50%	68.07	59.00	6.00	4.00	14.54	3.00	1.30	44.19	35.88
75%	79.00	67.00	6.43	5.00	18.60	6.66	1.60	60.09	47.28
max	88.00	70.00	9.00	6.00	21.38	7.76	10.00	98.40	79.28

Table S1 shows the descriptive statistics, namely mean, standard deviation, minimum value, 25% percentile (i.e., for protein 25% of the data are less than 63.18), 50% percentile, 75% percentile and maximum value of the dataset in this study (Entire dataset is presented in Table S3 at the end of this document). Protein, target moisture, moisture, ash, carbs, fat and fiber are the features/predictors in our ML framework. Hardness and Chewiness are targets to predict. Hardness exhibits a higher standard deviation than Chewiness.

S2) Hyper-parameters that are selected by GridSearch.

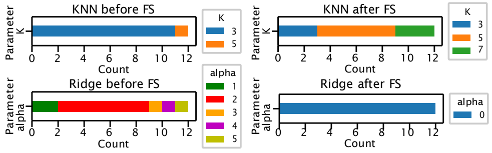

(a) KNN and Ridge.

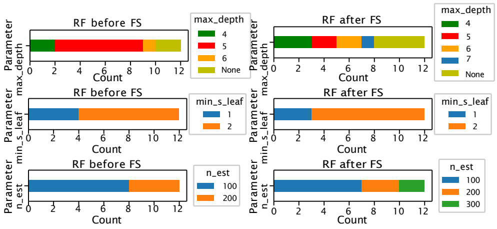

(b) Random Forest.

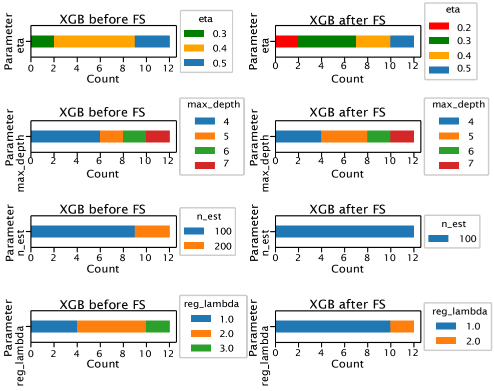

(c) XGBoost.

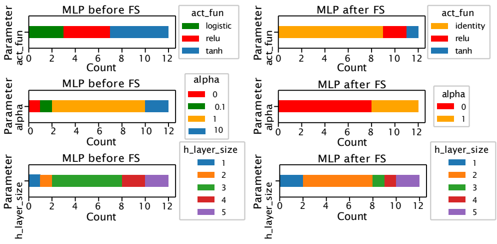

(d) Multilayer perceptron.

Figure S1: Hyper-parameters and their selection frequency during GridSearch across 12 folds. Selection frequency difference before and after the feature selection for Chewiness is shown.

Figure S1 shows the number of selections (selection frequency) of hyper-parameters across 12 folds during grid search with leave-one-group-out cross-validation and the effect of feature selection (FS) on hyper-parameter selection. After FS the necessity for regularization was dropped and models become more robust against overfitting. For instance, alpha and reg_lambda parameters which are L2 regularization parameters, exhibit a decrease on Ridge, MLP and XGBoost models. We also observed that less conservative values for model complexity parameters such as higher maximum depth of the tree was enabled in tree-based models, Random Forest and XGBoost, and higher K for KNN. Supplementary information S3 also supports that model's capability of generalization to unseen data is increased.

S3) Performance comparison between before and after feature selection.

Table S2 shows the performance comparison before and after feature selection. Subset “all features” represents full feature set before feature selection. We observe an average RMSE improvement of 60% and 67%; and an average MAPE improvement of 58% and 57% for Hardness and Chewiness, respectively.

Table S2: Textural characteristics prediction scores before and after feature selection.

	**Hardness**						**Chewiness**					
	**After Feature Selection (FS)**			**Before Feature Selection (FS)**			**After Feature Selection (FS)**			**Before Feature Selection (FS)**		
	**Subset**	**RMSE**	**MAPE %**	**Subset**	**RMSE**	**MAPE %**	**Subset**	**RMSE**	**MAPE %**	**Subset**	**RMSE**	**MAPE %**
Ridge	{target moisture, moisture, carbs, fat}	**10.101**	22.9	all features	78.190	118.8	{target moisture, moisture, carbs, fat}	**6.035**	14.5	all features	74.537	92.4
Random Forest	{protein, target moisture, carbs}	13.797	24.9	all features	14.462	32.9	{protein, target moisture, carbs, fat}	10.150	22.4	all features	11.030	25.8
XGBoost	{protein, carbs, fat, fiber}	12.310	21.2	all features	12.660	23.6	{protein, carbs, fat}	7.815	17.5	all features	7.908	16.8
KNN	{target moisture, moisture, ash, carbs, fat}	10.389	19.9	all features	14.298	26.1	{target moisture, carbs, fat}	7.902	16.1	all features	11.122	23.9
MLP	{target moisture, moisture, carbs, fat}	14.695	27.5	all features	33.930	75.8	{target moisture, moisture, carbs, fat}	8.018	16.3	all features	18.452	42.7
Average		12.258	23.28		30.708	55.44		7.984	17.36		24.610	40.32

S4) Entire dataset of the study. Entire dataset curated in this study is shown in Table S3.

Table S3: Curated dataset in this study.

Sample	protein	target moisture	moisture	ash	carbs	fat	fiber	Hardness	Chewiness	Group_ID	Study_ID	Meat analog type
1	62.699	56.850	5.394	4.245	20.491	6.662	0.510	46.709	38.050	5	2	extrusion_0%Wheat_Gluten
2	62.699	56.850	5.394	4.245	20.491	6.662	0.510	40.437	31.295	5	2	extrusion_0%Wheat_Gluten
3	62.699	57.160	5.394	4.245	20.491	6.662	0.510	49.060	39.495	5	2	extrusion_0%Wheat_Gluten
4	63.179	57.510	5.914	3.955	19.861	6.582	0.510	38.653	26.862	6	2	extrusion_10%Wheat_Gluten
5	63.179	57.240	5.914	3.955	19.861	6.582	0.510	50.812	38.977	6	2	extrusion_10%Wheat_Gluten
6	63.179	57.340	5.914	3.955	19.861	6.582	0.510	45.185	36.209	6	2	extrusion_10%Wheat_Gluten
7	63.659	56.600	6.434	3.665	19.231	6.502	0.510	54.000	36.175	7	2	extrusion_20%Wheat_Gluten
8	63.659	56.400	6.434	3.665	19.231	6.502	0.510	69.284	47.462	7	2	extrusion_20%Wheat_Gluten
9	63.659	56.450	6.434	3.665	19.231	6.502	0.510	59.359	41.746	7	2	extrusion_20%Wheat_Gluten
10	64.139	57.810	6.954	3.375	18.601	6.422	0.510	82.949	47.345	8	2	extrusion_30%Wheat_Gluten
11	64.139	57.210	6.954	3.375	18.601	6.422	0.510	77.025	47.472	8	2	extrusion_30%Wheat_Gluten
12	64.139	57.400	6.954	3.375	18.601	6.422	0.510	75.850	41.149	8	2	extrusion_30%Wheat_Gluten
13	72.171	53.111	5.315	4.291	10.444	7.758	0.020	98.401	79.282	9	3	elongation 40%Wheat Gluten
14	72.171	51.690	5.315	4.291	10.444	7.758	0.020	89.790	73.539	9	3	elongation 40%Wheat Gluten
15	72.171	56.331	5.315	4.291	10.444	7.758	0.020	91.139	74.830	9	3	elongation 40%Wheat Gluten
16	69.438	51.258	4.860	4.018	14.089	7.576	0.020	74.375	64.850	10	3	elongation 60%Wheat Gluten
17	69.438	54.251	4.860	4.018	14.089	7.576	0.020	70.018	58.413	10	3	elongation 60%Wheat Gluten
18	69.438	54.328	4.860	4.018	14.089	7.576	0.020	67.651	53.783	10	3	elongation 60%Wheat Gluten
19	66.704	53.697	4.404	3.745	17.733	7.393	0.020	69.774	58.305	11	3	elongation 80%Wheat Gluten
20	66.704	54.750	4.404	3.745	17.733	7.393	0.020	65.300	54.780	11	3	elongation 80%Wheat Gluten
21	66.704	53.319	4.404	3.745	17.733	7.393	0.020	65.460	55.666	11	3	elongation 80%Wheat Gluten
22	63.971	43.671	3.949	3.471	21.378	7.211	0.020	39.048	33.810	12	3	elongation 100%Wheat Gluten
23	63.971	48.689	3.949	3.471	21.378	7.211	0.020	48.827	41.327	12	3	elongation 100%Wheat Gluten
24	63.971	48.570	3.949	3.471	21.378	7.211	0.020	52.663	46.232	12	3	elongation 100%Wheat$_{G}luten$
25	79.000	66.000	6.000	4.000	9.200	0.200	1.600	21.844	19.110	1	1	Yellow pea isolate commercial (YPI-com)
26	79.000	66.000	6.000	4.000	9.200	0.200	1.600	24.833	21.570	1	1	Yellow pea isolate commercial (YPI-com)
27	79.000	66.000	6.000	4.000	9.200	0.200	1.600	26.284	23.069	1	1	Yellow pea isolate commercial (YPI-com)
28	79.000	67.000	6.000	4.000	9.200	0.200	1.600	23.412	19.806	1	1	Yellow pea isolate commercial (YPI-com)
29	79.000	67.000	6.000	4.000	9.200	0.200	1.600	17.875	15.386	1	1	Yellow pea isolate commercial (YPI-com)
30	79.000	67.000	6.000	4.000	9.200	0.200	1.600	20.884	17.816	1	1	Yellow pea isolate commercial (YPI-com)
31	79.000	68.000	6.000	4.000	9.200	0.200	1.600	19.463	19.463	1	1	Yellow pea isolate commercial (YPI-com)
32	79.000	68.000	6.000	4.000	9.200	0.200	1.600	17.385	15.131	1	1	Yellow pea isolate commercial (YPI-com)
33	79.000	68.000	6.000	4.000	9.200	0.200	1.600	17.248	14.935	1	1	Yellow pea isolate commercial (YPI-com)
34	79.000	69.000	6.000	4.000	9.200	0.200	1.600	18.914	16.092	1	1	Yellow pea isolate commercial (YPI-com)
35	79.000	69.000	6.000	4.000	9.200	0.200	1.600	19.757	17.493	1	1	Yellow pea isolate commercial (YPI-com)
36	79.000	69.000	6.000	4.000	9.200	0.200	1.600	23.843	20.335	1	1	Yellow pea isolate commercial (YPI-com)
37	79.000	70.000	6.000	4.000	9.200	0.200	1.600	14.631	12.338	1	1	Yellow pea isolate commercial (YPI-com)
38	79.000	70.000	6.000	4.000	9.200	0.200	1.600	16.680	14.171	1	1	Yellow pea isolate commercial (YPI-com)
39	79.000	70.000	6.000	4.000	9.200	0.200	1.600	23.775	19.874	1	1	Yellow pea isolate commercial (YPI-com)
40	81.000	67.000	2.000	5.000	7.600	3.000	1.600	21.335	17.689	2	1	Yellow pea isolate local (YPI-local)
41	81.000	67.000	2.000	5.000	7.600	3.000	1.600	22.070	17.601	2	1	Yellow pea isolate local (YPI-local)
42	56.000	58.000	9.000	6.000	15.000	3.000	10.000	60.133	50.813	3	1	Faba bean concentrate commercial (FBC-com)
43	56.000	58.000	9.000	6.000	15.000	3.000	10.000	59.976	51.587	3	1	Faba bean concentrate commercial (FBC-com)
44	56.000	58.000	9.000	6.000	15.000	3.000	10.000	45.443	39.582	3	1	Faba bean concentrate commercial (FBC-com)
45	56.000	60.000	9.000	6.000	15.000	3.000	10.000	53.655	47.099	3	1	Faba bean concentrate commercial (FBC-com)
46	56.000	60.000	9.000	6.000	15.000	3.000	10.000	44.061	38.357	3	1	Faba bean concentrate commercial (FBC-com)
47	56.000	60.000	9.000	6.000	15.000	3.000	10.000	51.176	43.316	3	1	Faba bean concentrate commercial (FBC-com)
48	56.000	62.000	9.000	6.000	15.000	3.000	10.000	37.044	29.420	3	1	Faba bean concentrate commercial (FBC-com)
49	56.000	62.000	9.000	6.000	15.000	3.000	10.000	44.316	35.584	3	1	Faba bean concentrate commercial (FBC-com)
50	56.000	62.000	9.000	6.000	15.000	3.000	10.000	39.288	32.722	3	1	Faba bean concentrate commercial (FBC-com)
51	88.000	62.000	2.000	5.000	2.900	0.300	1.300	25.950	22.628	4	1	Faba bean isolate local (FBI-local)
52	88.000	64.000	2.000	5.000	2.900	0.300	1.300	32.350	27.518	4	1	Faba bean isolate local (FBI-local)
53	88.000	66.000	2.000	5.000	2.900	0.300	1.300	34.535	29.322	4	1	Faba bean isolate local (FBI-local)
54	88.000	70.000	2.000	5.000	2.900	0.300	1.300	25.264	20.854	4	1	Faba bean isolate local (FBI-local)
